# Current Applications of Gas Sensor Based on 2-D Nanomaterial: A Mini Review

**DOI:** 10.3389/fchem.2019.00839

**Published:** 2019-12-10

**Authors:** Liang Ge, Xiaolin Mu, Guiyun Tian, Qi Huang, Junaid Ahmed, Ze Hu

**Affiliations:** ^1^Electrical and Mechanical Engineering Department, Southwest Petroleum University, Chengdu, China; ^2^Department of Engineering, Newcastle University, Newcastle upon Tyne, United Kingdom; ^3^Electrical Department, Sukkur Institute of Business Administration, Sukkur, Pakistan

**Keywords:** 2-D nanomaterial, gas sensor, performance improvement, current application, development trend

## Abstract

Gas sensor, as one of the most important devices to detect noxious gases, provides a vital way to monitor the concentration and environmental information of gas in order to guarantee the safety of production. Therefore, researches on high sensitivity, high selectivity, and high stability have become hot issues. Since the discovery of the nanomaterial, it has been increasingly applied to the gas sensor for its distinguishing surface performances. However, 0-D and 1-D nanomaterials, with limited electronic confinement effect and surface effect, cannot reach the requirement for the production of gas sensors. This paper gives an introduction about the current researching progress and development trend of 2-D nanomaterials, analyzes the common forms of 2-D nanoscale structure, and summarizes the mechanism of gas sensing. Then, widely concerned factors including morphological properties and crystalline structure of 2-D nanomaterial, impact of doped metal on the sensibility of gas sensors, impact of symmetry, and working temperature on the selectivity of gas sensors have been demonstrated in detail. In all, the detailed analysis above has pointed out a way for the development of new 2-D nanomaterial and enhancing the sensibility of gas sensors.

## Introduction

Nanotechnology, a newly developed technology based on quantum mechanics, molecular biology, material science, microelectronics, and computer technology, is a scientific way to synthesize new materials on nanoscales. Prof. Tanggulachi firstly defined this newly emerged subject as nanotechnology in 1974 and clarified that the research on the characteristics and applications of nanomaterial should be restricted to the scale of 0.1–100 nm (Zhu et al., 2010; Huang et al., 2014). Nanomaterial has dramatic advantages over traditional material. Those advantages include distinguishing surface effect (Zhang, 2011) and quantum size effect (Xu et al., 2019). Factors like tiny particles, large surface areas, and high surface energy will enhance the performances of nanomaterial tremendously (Wang, 2013).

Gas sensor (You et al., 2012) converts the components and concentrations of various gases into standard electrical signals by using specific physical and chemical effects. It has been widely used in the detection of noxious and harmful gases and natural gas leakage. It has been improved greatly since the 1970s, with nanomaterials changed from single metallic oxide to combined metal oxide. Large progress has also been made on the sensing performances like sensitivity, accuracy, and stability when detecting specific variety of gas (Gonullu et al., 2012; Trung et al., 2012; Xu et al., 2012).

In recent years, the development of electronic devices is more about integration, miniaturization, and even microminiaturization. Nanomaterial plays an increasingly important role in the improvement of gas sensors. Based on the gas-sensing properties of 2-D nanomaterials such as response speed, selectivity, and stability, this paper gives a review of the factors that influence the performance of 2-D nanomaterial gas sensors and proposes the future development trend of the improvement of these sensors' parameters.

## Development of Research on 2-D Nanomaterial

Since the successful extraction of graphene (a 2-D nanoscale graphite with single atomic layer) by the Geim Group (Geim and Novoselov, 2007; Geim, 2009; Perreault et al., 2015; Varghese et al., 2015) from Manchester University, UK, in 2004, more and more researchers have been attracted to the study of 2-D nanomaterial. According to numerous researches, nanomaterials are sorted into four categories by their number of nanoscale dimension. Each nanomaterial has different gas sensitivities due to electronics confinement effect, surface effect, etc. Generally speaking, three dimensions of 0-D nanomaterial are all in the scale of nanometers. Those 0-D nanomaterials include nanoscale particles, metallic cluster, etc. 1-D nanomaterial has two dimensions in nanoscale, with the other one of non-nanoscale size, such as the organic chain structures of nanoscale tube (Ma et al., 2014), nanoscale line (Tao et al., 2011), nanoscale band (Sun et al., 2003), nanoscale rod (Zhang et al., 2003), etc. 2-D nanomaterial only has one dimension of nanoscale, such as nanoscale membrane, 2-D single layer structure (Li et al., 2009), and nanoscale sheets (Liu et al., 2016). 3-D nanomaterial, such as nanoscale flowers (Wang and Rogach, 2014), etc. can be sorted into organic and inorganic nanomaterials according to its components.

**Figure 1:**
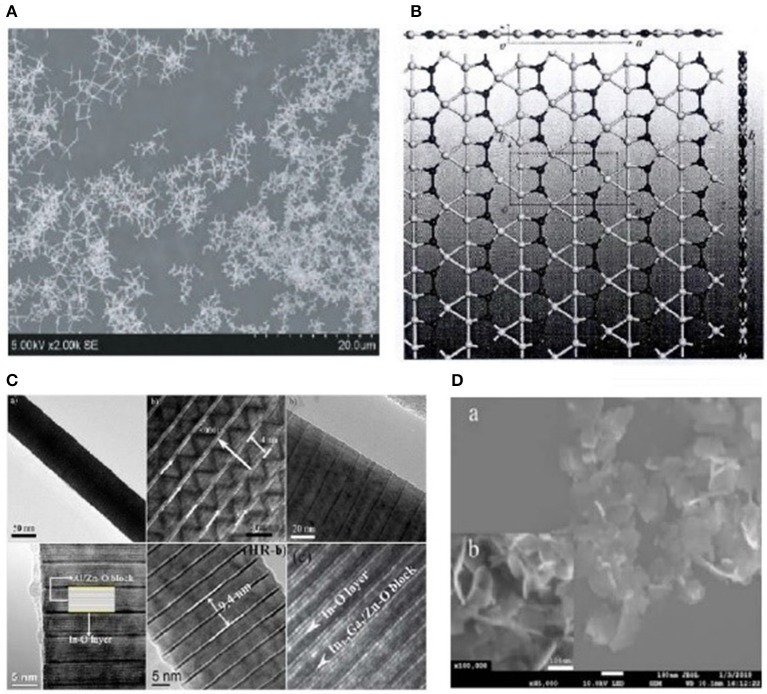
Electron microscopic images of partial 2-D nanomaterials. **(A)** ZnO nanoscale membrane (adapted from Shen et al., 2018 with permission from Shen). **(B)** 2-D B_3_N_2_ single-layer structure (adapted from Sun et al., 2016 with permission from Sun). **(C)** InMO_3_(ZnO)_m_ superlattice (adapted from Wang, 2018 with permission from Wang). **(D)** New SnO_2_ nanoscale sheets (adapted from Yue and Yu, 2019 with permission from Yue).

In 2000, Kong et al. proved that a 1-D carbon nanoscale tube (CNTs) can detect the existence of NH_3_ and NO_2_ at low concentration under room temperature. CNTs have high absorption efficiency with rich adsorption structures and adsorption points, and they also have great value when it comes to application (Kong et al., 2000). In 2009, based on 2-D nanomaterial, Zhang et al. developed the SnO_2_ hollow microsphere, which was used for NO_2_ sensor. The results showed that SnO_2_ hollow sphere sensors can respond to NO_2_ at ppm level under 160°C and distinguishing selectivity (Zhang et al., 2009). In 2013, Sharm et al. produced a WO_3_ cluster/tin oxide heterostructure that can detect NO_2_ with low concentration of 10 ppm under 100°C (Sharma et al., 2013). In 2014, Rumyantsev et al. synthesized 2-D thin-film transistor MoS_2_ as well as figured out the CV graph of it. Comparing this MoS_2_ with ethanol, hexanenitrile, toluene, chloroform, and methanol on a time–current graph, a conclusion can be drawn that this material had better selectivity to alcohols (Shur et al., 2014). By using MoS_2_ layers of different thicknesses, photodetection of gas could be achieved (Wen et al., 2018). In 2016, Pang et al. used CNTs doped with nanoscale SnO_2_ particles to produce formic acid gas sensors, with their sensitivity reaching 13.49 (Pang et al., 2016). Also, this material lowered the working temperature by 120°C and shortened the response time by 4 s. Sun et al. constructed a graphene-like single-layered nanoscale structure (shown in Figure 1B). Though this material was a non-magnetic conductor, it had metallic characteristics. It could be a new research material in the nanotechnology field (Sun et al., 2016). In 2017, Tao et al. used ultrasonic spray pyrolysis with electrostatic enhancement to produce 2-DMWCNTs/SnO_2_ nanocomposite. When the deposition temperature was 300°C with MWCNTs' doping amount reaching 10 mg/ml, the performances of gas-sensitive material have improved greatly (Tao, 2017). In 2017, Yuan obtained 2-D PS/WO_3_ hollow nanoscale gas sensor with thick membrane by spin coating WO_3_ hollow nanoscale structure on the surface of PS. The experiment showed that this material had high sensitivity and distinguishing response characteristics when facing ppb level of NO_2_ (Yuan, 2017). In 2018, in order to cope with the problems that pure phase α − MoO_3_ had (excessively high working temperature, low stability, and long response time), Yu synthesized 2-DTiO_2_/α − MoO_3_ nanosheet, α − MoO_3_ − PANI p − n heterojuction, Au/α − MoO_3_ composite nanosheet, etc., which improved its sensitivity and stability greatly and shortened the response time (Yu, 2018). Shen et al. applied chemical vapor deposition (CVD) to synthesize ZnO nanofilm on the glass substrate, with the material having the best sensitivity to ethanol at room temperature (shown in Figure 1A) (Shen et al., 2018). Wang et al. composed Zn-Sn-O superlattice nanoscale particle (shown in Figure 1C), which had good selectivity and extremely high sensitivity to H_2_S (Wang, 2018). In 2019, Yu et al. made a new SnO_2_ nanoscale sheet structure (shown in Figure 1D), with the sensitivity of 12.14 at its best working temperature of 175°C and concentration of 100 ppm. This nanomaterial showed a tremendous improvement of sensitivity to gas like ethanol and formaldehyde (Yue and Yu, 2019). Kou's group systematically demonstrated the electronic, structure, and transport characters of monolayer BP with the adsorption of several typical gas molecules, CO, NH_3_, CO, NO_2_, and NO (Liu and Zhou, 2019). In 2018, Qiu et al. used ultraviolet rays and ozone method to *in-situ* synthesize oxide graphene membrane. The gas sensor of high performance can be made by combining oxide graphene membrane and two-terminal electrical devices. This sensor has higher sensitivity to NH3, and better selectivity to NH3 compared with acetone and absolute ethanol (Qiu et al., 2018). In 2019, Yang's study for dissertation proved this conclusion (Yang, 2019). Guo developed graphene/polyaniline material and analyzed its gas-sensing performances to multiple gases including NH_3_, CO, NO, H_2_, etc. The results showed that this material is characterized by higher selectivity to NH_3_ because of higher sensitivity and stronger adsorption to it (Guo, 2018).

## Gas-Sensing Performance of Gas Sensor Based on 2-D Nanomaterial

### Gas-Sensing Mechanism Based on 2-D Nanomaterial

The detecting principle of gas sensor is that gas molecules are adsorbed on the surface of the substrate nanomaterial. Then, charge transfer occurs between the gas molecule and the substrate material, which changes the resistivity of the substrate nanomaterial. By testing the resistivity of the substrate nanomaterial, characteristics of gas such as properties and concentration can be known. When it comes to substrate nanomaterial, process of choosing from 0-D quantum dot, 1-D quantum wire, to 2-D quantum surface is experienced. A large number of studies have shown that 2-D nanomaterial has larger surface compared with 0-D and 1-D nanomaterials. Special membranous or lamellar structure has stronger capability to absorb gas molecules. Meanwhile the gas sensitivity can be improved by metal doping to pure nanomaterial (Dai and Yuan, 2010; Beheshtian et al., 2012a,b; Zhang et al., 2012; Rastegar et al., 2013; Ahmad et al., 2019; Choi et al., 2019; Kim et al., 2019). Take graphene for instance. Pure graphene absorbs common gas molecules physically, which has a large limitation on gas sensitivity (Feng et al., 2012; Meng et al., 2013; Abideen et al., 2018; Mirzaei et al., 2019; Mourya et al., 2019). The performance of graphene can be changed by metal doping. Arsenene 2-D semiconductor structure (Fleurence et al., 2012), antimony-vinyl folded honeycomb 2-D structure, and telluriene structure can be produced by imitating the graphene's structure (Liu, 2017). In conclusion, 2-D nanomaterial has higher sensitivity and selectivity when compared with other materials on gas-sensing mechanism.

### Effect of 2-D Nanomaterial on Gas-Sensing Performance

Gas-sensitive materials have many properties, such as sensitivity, selectivity, stability, response time, etc., which are directly related to the surface characteristics of material. Surface characteristics are decided by the particle size of material. When the scale of the material reaches nano size, its surface area and surface activity will increase. Nanomaterials can be designed into different shapes, which will greatly increase the capability of absorbing specific gases. Therefore, gas sensors made by those nanomaterials will have their performances enhanced dramatically. Take SnO_2_ as an example; the porous hollow rod SnO_2_ composed of 1-D nanoscale rod provides more paths for gaseous diffusion, due to massive petal shape nanosheets and pores. The conversion of sphere-like structures into petal-like ones and successful synthesis of 1-D nanoscale rod and cones when composing flower-like SnO_2_ improves the gas sensitivity and shortens the response time. In order to avoid the performance degradation caused by accumulation of nanostructure, the porous flower-like SnO_2_ structure composed by 2-D nanosheets not only enlarges the surface area of the structure but also increases the internal hole channels as well, which will promote gas diffusion. Finally, flake-like layered SnO_2_ structure composed by numerous thin nanosheets marks further improvement on the material's surface activity and shows excellent gas selectivity and sensitivity in the test (shown in Table 1).

**Table 1:** Relationship between microstructure, preparation, and gas sensitivity in 2-D nanomaterial (WO_3_, ZnO).

**Gas-sensitive material**	**Operation temperature (**°**C)**	**Target gas**	**Detection range (ppm)**	**The dynamic responses R**		**Response time (s)**	**References**
				**R = Ra/Rg**	**Concentration (ppm)**		
WO_3_ nanoscale sheet	150	NO_2_	1–20	107.3	5.00	–	Qin et al., 2010
WO_3_ hollow half tube	300	H_2_S	0.12–2	1.2	0.12	35	Choi et al., 2012
ZnO nanoscale sheet	350	Ethanol	1–500	20.0	100.00	12	Alenezi et al., 2013
WO_3_ hollow crystal sheet	340	Ethanol	10–500	2.5	10.00	–	Su et al., 2010
WO_3_ hollow microshpere	75	NO_2_	0.04–1	16.0	0.04	10	You et al., 2012
WO_3_ nanoscale cluster	320	Acetone	1–400	17.5	100.00	2	Huang et al., 2011
ZnO flower structure	370	Ethanol	5–500	31.0	100.00	12	Chen et al., 2013
ZnO nest	420	Acetone	5–1,000	17.4	100.00	7	Wang et al., 2012

### Effect of 2-D Nanomaterial on Gas Sensor's Sensitivity

Improving the response sensitivity of nanomaterial gas sensor is crucial in practical engineering applications. Response sensitivity can be improved by (1) changing the surface of the 2-D nanomaterial (generally metallic oxide) to enhance the sensibility of the reaction 1-D nanofiber formed by nanoparticles will provide more route for the electron to move rapidly and bigger specific surface area to improve its sensibility; Cho et al. produced hemispherical N_i_O nanomaterial, which had a sensitivity of 1.5 to ethanol vapor (Cho et al., 2011); Song et al. produced nanotube N_i_O nanomaterial, with a sensitivity of 3 to ethanol vapor; the particle size and touching area are also the important factors on the sensitivity (Song et al., 2011), (2) changing the morphology of the nanomaterial Szilagyi et al. sintered ammonium tungstate compounds to produce hexagonal phase h-WO_3_, which was sensitive to H_2_S and monoclinic γ − WO_3_, CH_4_, H_2_, and CO (Szilagyi et al., 2010); Gao et al. used hydrothermal method to synthesize triclinic δ − WO_3_ square nanosheet, which had higher gas response sensitivity to cyclohexene (Gao et al., 2013), and (3) doping of transition metal to improve the sensitivity of nanomaterial Wang et al. doped Cr to produce ferroelectric monoclinic ε − WO_3_, which had better selectivity to acetone (Wang et al., 2008); Kim et al. prepared N_i_O doped with Fe^3+^ to make a gas sensor with the response value of 100 ppm ethanol improved from 5.5 to 172.5, which showed tremendous improvement of nanomaterial sensitivity by metallic doping (Kim, 2012).

### Effect of 2-D Nanomaterial on Gas Sensor Selectivity

Something interesting will be found when we combine special microstructure of nanomaterial with specific structure of different gases. This interesting finding will be the nanomaterial's specific selectivity to gases in macroscopic. Selectivity of 2-D nanomaterial is an important factor to measure the effect of materials. The lower symmetry of structure indicates the better selectivity of gas sensors. Take WO_3_ nanocrystal with exocentric structure as an example. The symmetry of triclinic WO_3_ crystal is lower than that of monoclinic and hexagonal WO_3_ crystals. So, the triclinic WO3 crystal has higher sensitivity and selectivity on acetone molecules with larger dipole moments (Bai, 2014). The influence of working temperature on the selectivity of materials is also an important factor to consider. Triclinic WO_3_ nanomaterial has better selectivity and sensitivity to acetone at higher temperature and better selectivity to NO_2_ at lower temperature (Zhao et al., 2013).

## Development Trend of Gas Sensor Based on 2-D Nanomaterial

Throughout the development of nanotechnology, gas sensor based on nanomaterial is always an extremely important research field. It has deep and wide influence on life and production. However, 2-D nanomaterial research is still full of problems in the aspects ranging from imperfect sensitive materials and immature preparation technology to disability on scaled production. Gas sensor materials will be developed from single metallic oxide to composite oxide. Morphology of nanomaterial can be changed to improve the sensing performances; particle size can be reduced to improve the surface activity of the material; new structure can be designed to absorb more specific gases; selectivity of the sensors can be improved by reducing the asymmetry of 2-D nanomaterial structure and improving its working temperature.

2-D nanomaterial plays an increasingly important role in the further improvement of the gas sensor's performance. In recent years, the development trend of various electronic components tends to be more integrated, miniaturized, and even microminiaturized. Gas sensor will also consume less power, be multi-functional, and have higher performance. The material of gas sensor will be changed from simple gas-sensitive materials to complex composite materials. The structure of gas sensor will be changed from monolayer to multilayer and from simple morphology to special morphology. Also, it will be widely used in chemical production, gas transportation, and other toxic and harmful gas detection. In 2019, Tian et al. prepared ternary complex of graphene/WO3 nanorod/polythiophene (3D-r GO/WO3/PTh), and studied its gas sensitivity to H2S. The study results showed that under low temperature of 75°C, this material has fast response and distinguishing selectivity to H2S (Tian et al., 2019). Therefore, it can be found that the study of multiple element compound is a very popular research issue for the development of new gas sensor materials with better performance.

## Conclusion

In this paper, gas-sensing properties of the 2-D nanomaterial are reviewed. Firstly, the classification of nanomaterials on the number of dimensions is briefly introduced, and the latest research progress and development trend of 2-D nanomaterial are summarized. Secondly, the gas-sensing mechanism of 2-D nanomaterial is summarized by comparing the characteristics of existing 2-D nanomaterials. The effects of particle size and morphology property of 2-D nanomaterial on the performance of gas sensors are discussed. Then, the enhancement related to morphology property, phase structure, and metal doping of 2-D nanomaterial on the sensitivity of gas sensors is analyzed. Also, the effect of symmetry structure of 2-D nanomaterial on the selectivity of gas sensors is concluded. Finally, the development trend of 2-D nanomaterials for gas sensors is proposed, and references for the next development of 2-D nanomaterial are provided.

## Author Contributions

XM and LG conceived and designed the experiments. ZH and GT performed the experiments. XM and JA analyzed the data. XM and QH wrote the manuscript with input from all authors. All authors read and approved the manuscript.

### Conflict of Interest

The authors declare that the research was conducted in the absence of any commercial or financial relationships that could be construed as a potential conflict of interest.
